# A simple pooling method for variable selection in multiply imputed datasets outperformed complex methods

**DOI:** 10.1186/s12874-022-01693-8

**Published:** 2022-08-04

**Authors:** A. M. Panken, M. W. Heymans

**Affiliations:** 1 Department of Epidemiology and Data Science, Amsterdam UMC, Vrije Universiteit Amsterdam, Amsterdam Public Health Research Institute, Amsterdam, The Netherlands; 2 Physical Therapy Practice Panken, Roermond, The Netherlands

**Keywords:** Logistic regression, Median-p-rule, Multiple imputation, Pooling selection methods, Variable selection

## Abstract

**Background:**

For the development of prognostic models, after multiple imputation, variable selection is advised to be applied from the pooled model. The aim of this study is to evaluate by using a simulation study and practical data example the performance of four different pooling methods for variable selection in multiple imputed datasets. These methods are the D1, D2, D3 and recently extended Median-P-Rule (MPR) for categorical, dichotomous, and continuous variables in logistic regression models.

**Methods:**

Four datasets (*n* = 200 and *n* = 500), with 9 variables and correlations of respectively 0.2 and 0.6 between these variables, were simulated. These datasets included 2 categorical and 2 continuous variables with 20% missing at random data. Multiple Imputation (m = 5) was applied, and the four methods were compared with selection from the full model (without missing data). The same analyzes were repeated in five multiply imputed real-world datasets (NHANES) (m = 5, *p* = 0.05, *N* = 250/300/400/500/1000).

**Results:**

In the simulated datasets, the differences between the pooling methods were most evident in the smaller datasets. The MPR performed equal to all other pooling methods for the selection frequency, as well as for the P-values of the continuous and dichotomous variables, however the MPR performed consistently better for pooling and selecting categorical variables in multiply imputed datasets and also regarding the stability of the selected prognostic models. Analyzes in the NHANES-dataset showed that all methods mostly selected the same models. Compared to each other however, the D2-method seemed to be the least sensitive and the MPR the most sensitive, most simple, and easy method to apply.

**Conclusions:**

Considering that MPR is the most simple and easy pooling method to use for epidemiologists and applied researchers, we carefully recommend using the MPR-method to pool categorical variables with more than two levels after Multiple Imputation in combination with Backward Selection-procedures (BWS). Because MPR never performed worse than the other methods in continuous and dichotomous variables we also advice to use MPR in these types of variables.

## Background

Logistic regression analysis is a widely used method in epidemiological and medical studies for developing prognostic models. Although researchers try to avoid it, missing data occur in many study designs. The most recommended method for processing incomplete data is Multiple Imputation (MI) [1]; this method is nowadays integrated in almost all regular statistical software packages and is therefore within reach of many researchers. MI generates multiple imputed datasets, after which a complete data analysis can be applied in each imputed dataset. Finally, parameter estimates can be combined using Rubin’s Rules (RR) [2]. If a dataset contains missing values, it is recommended to apply MI before excluding variables in logistic regression with backward selection (BWS) from the pooled model [3–6]. RR can be used to calculate pooled coefficients and standard errors (SE’s). This is easy to apply for continuous and dichotomous variables where a single Wald statistic is used to calculate a P-value to determine significance [7]. This is complex for categorical variables with more than two levels. For variable selection with categorical variables several methods have been developed [1, 7]: 1. Method D1 (Multivariate Wald test): this test pools within and between covariance matrices across imputed datasets. After that, the total parameter covariance matrix of the multivariate Wald test is corrected to account for the missing data [1, 8]. 2. Method D2 (Pooling Chi-square statistics): this test uses the chi-square values from the multiple parameter Wald or likelihood ratio tests across imputed datasets and pools them [9]. 3. Method D3 (Combining Likelihood Ratio statistics): this test is based on pooled likelihood ratio statistics and requires fitting multiple models across imputed datasets for each categorical variable in the data and can therefore be a very time-consuming process [10, 11]. A disadvantage is that none of these methods is integrated in regular statistical software packages for variable selection when developing prognostic models. Also, these methods do not always obtain the optimal P-values, so selection of the correct variables is not guaranteed [10]. Therefore, researchers sometimes opt for a single imputation, often leading to an incorrect selection of variables [12].

Van de Wiel et al. introduced the median of the P-values (MPR) as method to compare prognostic models [13]. Eekhout et al. compared MPR with the pooling methods D1, D2, D3 in MI-datasets in a simulation study. They found that the MPR was an attractive rule for statistical inference of categorical variables with more than two levels because it showed to have equal power as the D1-, D2- and D3-method but was much easier to apply in any software package [1].

The MPR method may therefore be a potential attractive method for variable selection including models with categorical variables. Until now, these methods have never been compared to derive a prognostic model in logistic regression models after MI.

Therefore, the aim of this study is to evaluate four different pooling methods for variable selection in Multiply Imputed datasets for categorical, dichotomous, and continuous variables in a logistic regression with a backward selection (BWS) procedure. The selection frequency of the variables, the P-values of the pooled selection results and the stability of the models in Multiply Imputed datasets will be compared with the results from the BWS-procedure in the complete dataset (without missing data). All analyzes will be repeated in a real-world dataset.

## Method

To evaluate the results of the four different pooling methods in Multiply Imputed datasets after a BWS-procedure, we conducted a simulation study and repeated the procedures in a real-world dataset (NHANES).

### Simulation datasets

- 1) To generate simulated datasets, we used as input parameters the mean values and the standard deviations (SD) of the variables of an empirical dataset of low back pain patients [14]. See Table 1. A total of 9 variables were drawn from a multivariate normal distribution using the mean and SD as input parameters.
- 2) A set of 9 variables, including categorical, dichotomous, and continuous variables (normally distributed) was generated.
- 3) In the simulated datasets, categorical and dichotomous variables were initially considered to be continuous to determine their level of correlation and were then subsequently categorized (in 4 categories) and dichotomized by using cutoff values from the empirical dataset. For the categorical variables ‘Cat1’ and ‘Cat2’ the cutoff values (in percentages) were: [0, 0.6, 0.8, 0.9, 1] and [0, 0.3, 0.6, 0.8, 1]. For both dichotomous variables the median was used as a cutoff value.
- 4) The outcome measure was obtained by first calculating the linear predictor score by multiplying coefficients from the empirical dataset (Table 1) by the predictor values. A logistic regression model was then used to convert these scores into probabilities and a uniform distribution to convert these into a binary outcome, according to the rule that when the probability is lower than the value from the uniform distribution the outcome is 1 and 0 otherwise.
- 5) Four different datasets were simulated. Two sets with 200 observations, one with a correlation degree of 0.2 and the other with a degree of 0.6 and similarly two sets with 500 observations.
- 6) One of the regression coefficients in the model had an effect size of zero to mimic the behavior of a noise variable during variable selection (‘Noise’).

**Table 1:** Means and variances used for the simulated dataset

	Varname	Coefficients	Mean	Variance	Standard Deviation (SD)	Distribution
Cat1	Xcat1	0.5 / 1.5 / 1.5^a^	2	0.9	0.95	Normal
Cat2	Xcat2	1.5 / 1.5 / 1.5^a^	3	1	1	Normal
Dich1	X1	-0.5	0.8	0.2	0.45	Normal
Dich2	X2	-1	0.7	0.3	0.55	Normal
Cont1	X3	0.5	7	2	1.41	Normal
Noise	X4	0	7	2	1.41	Normal
Cont2	X5	-0.1	40	90	9.5	Normal
Cont3	X6	-0.1	26	15	3.9	Normal
Cont4	X7	-0.1	34	23	4.8	Normal

*Cat1 *Categorical variable 1, *Cat2* Categorical variable 2, *Dich1* Dichotomous variable 1, *Dich2* Dichotomous variable 2

*Cont1* Continuous variable 1, *Cont2* Continuous variable 2, *Cont3* Continuous variable 3, *Cont4* Continuous variable 4, *Noise* Noise variable

^a^Coefficients belonging to the dummies of the categorical variable

### Generating Missing data

Multiple Imputation is indicated under the missing at random (MAR) mechanism, which means that missing data can be covered by observed data [11]. In each of the four simulated datasets, 20 percent missing data were created in both categorical variables and two continuous variables (‘Noise’ and ‘Cont4’). To create these missing data, the missing at random (according the MAR) mechanism has been used. This means that the probability for missing data in the two categorical and continuous variables was related to other variables in the dataset. For this, data were made missing in each variable as a function of another variable (e.g., X1 =  X2 * 0.4 and X1 =   X2 * 0.167), to create a realistic data situation. We used the commands ‘defMiss’ (for defining the missing data matrix with the formula option) and ‘genMiss’ in R software package “simstudy”. These formula values were chosen in such a way that 20% of the data in each variable was missing and around 50% of the cases in each simulated sample.

### Imputation method

MI was performed in each simulated dataset generating 5 imputed datasets using Multivariate Imputation by Chained Equations (MICE) including the outcome in the imputation model [15, 16].

### Pooling methods

Four different pooling methods were used:

- 1) The pooled sampling variance method (D1), which contains a combination of the pooled parameter estimates and the pooled sampling variances of each imputed dataset to construct a test that resembles a multivariate Wald test [8, 17].
- 2) Multiple parameter Wald test (D2) which pools the chi-square values from the multiple parameter Wald or likelihood ratio tests [9].
- 3) Meng and Rubin pooling method (D3) which pools likelihood ratio tests [10].
- 4) The median-P-rule (MPR) which uses the median *P*-value of the significance tests conducted in each imputed dataset. Hence, it depends on *P*-values only and not on the parameter estimates [13].

### Statistics and analyses

Logistic regression analyses were performed in all original complete simulated datasets and in all multiply imputed datasets. The coefficients, SE’s, *P*-values, and all developed prognostic models were compared. The BWS-procedures were performed using different *P*-out selection values (between 1.0 and 0.05) to develop parsimonious prediction models containing the strongest prognostic variables as well as larger prediction models containing strong and less strong prognostic variables.

The entire procedure was repeated 500 times and the results were compared with the results of the BWS-procedure in the complete dataset (no missing data). All statistic procedures were performed in R.

### Comparing pooling methods

When comparing the pooling methods for variable selection, the focus was on three points:

### The selection frequency of the variables

The selection frequency of the variables of each method was compared with that of the complete datasets, i.e., without missing values, which served as a reference model. The frequency was obtained by summing up how many times a variable was selected in a model divided by the total number of simulated models within that run. Subsequently it was evaluated which pooling method showed the most similar selection frequencies compared to those in the complete dataset.

### The *P*-values of the selected variables

To compare the *P*-values, all *P*-values were first naturally log-transformed to be able to make even the smallest differences in *P*-values graphically visible. The median of these *P*-values of the four pooling methods was compared with the median of the *P*-values in the complete dataset.

### The stability of the selected prognostic models

Model stability was evaluated by providing model selection frequencies to quantify how many times a particular set of prognostic variables was selected [4, 18]. The first 10 unique prognostic models were evaluated for all pooling methods and for the models selected in the complete dataset. For example: In one simulation run 500 initial models were fitted by applying BWS and from these 500 models, 10 unique models in the complete dataset were selected. With method D1, 375 models were identical to those 10 unique models in the complete dataset and so counted for 375/500 = 75 percent of the same unique models. With method D2 we saw 350 unique models (70 percent). D3 showed 340 (68 percent) and MPR 450 (90 percent) the same unique models. In this example, MPR was the most stable pooling method. This way of analyzing the stability of the prognostic models was executed for all BWS-procedures under all different conditions.

### Analyses in the NHANES-dataset

The NHANES-dataset was used as a real-world data set to evaluate the performance of the same four pooling methods (D1, D2, D3 and MPR). Twelve variables were analyzed of which 6 continuous, 2 dichotomous and 4 categorical variables, with a dichotomous variable as outcome measure. MI was performed, generating 5 imputed datasets (m = 5), using Multivariate Imputation by Chained Equations (MICE) and including the outcome in the imputation model [15, 16]. A BWS-procedure was conducted with *P*-out < 0.05 in all pooling methods. The selected variables and developed models were compared.

### Software

To generate complete simulation data we used the R package “simstudy”, that was also used to generate the missing data (functions ‘defMiss’ and ‘genMiss’). Backward selection in the complete data was conducted with the package “rms” (function fastbw), imputations were done using the “mice” package and pooling with the “psfmi” package.

## Results

### The selection frequency of the variables

**Fig. 1:**
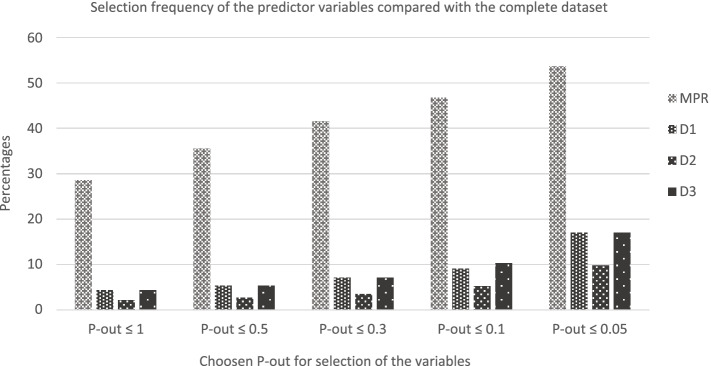
The selection frequency of the variables using different *P*-out-values compared to the complete dataset

What can be determined from Fig. 1 is that in general the MPR method performed better than the other selection methods when the P-out values became stricter. With a *p*-out ≤ 0.3 in 41.6 percent of the simulated samples, the MPR selected the same variables as in the complete dataset. The D1, D2 and D3 methods in only respectively 7.1, 3.5 and 7.1 percent of the cases. Using a stricter *p*-out (0.1) the MPR method selected the same variables as in the complete dataset in 46.8 percent of the cases and with a *P*-out of 0.05 in 53.7 percent of the cases. Using less strict *P*-out values (1.0 and 0.5) in 60.5 percent and 51 percent of the cases all pooling methods performed similarly.

Table 2 shows for all pooling methods the selection frequencies (percentages) of variables after BWS in Multiply Imputed datasets compared to the complete dataset. Categorical variables that were selected with the MPR-method were closest to those in the complete dataset. For the continuous and dichotomous variables none of the four pooling methods showed a better selection frequency compared to the complete dataset. Therefore, the MPR was the best pooling method to select prognostic variables. The noise variable had a very low selection frequency for all methods and was selected in less than 12 percent of the cases using a *P*-out ≤ 0.1 and 6 percent in models with a *P*-out ≤ 0.05.

**Table 2:** Percentages selection frequency of variables after backward selection in Multiply Imputed datasets using four different pooling methods and in the complete dataset

Dataset	Variable	D1	D2	MR	MPR	Comp*
*N* = 200. corr 0.2 *P*-out < 0.1	Noise	12.2	12.2	12.4	11.4^#^	11.4
	Cont4	63.8	64.6	64.6	67^#^	79.6
	Cat1	63.8	66.6	67.2	84.2^#^	87
	Cat2	76.6	83	84.8	92.8^#^	95.4
	Dich1	35.8	35.8	36.2	37.6^#^	38.2
*N* = 200. corr 0.6 *P*-out < 0.1	Noise	11.2^#^	11.4	11.2^#^	11.8	10.2
	Cont4	49.8	51.6	51.6	52.8^#^	65.2
	Cat1	51.8	51.8	52.4	73.6^#^	74.4
	Cat2	76.8	79.6	82	86.2^#^	86.8
	Dich1	32.4	33	32.8	34.6^#^	34.6
*N* = 200. corr 0.2 *P*-out < 0.05	Noise	6^#^	6.6	6.2	6.2	5.2
	Cont4	5	50.6	50.6	52.8^#^	67.2
	Cat1	53.2	54.6	54.2	74.6^#^	75.2
	Cat2	65	68.8	73	88^#^	86.2
	Dich1	27.2	27.2	27.4^#^	29	27.8
*N* = 200. corr 0.6 *P*-out < 0.05	Noise	6.4	6.8	6.2^#^	6.2^#^	4.8
	Cont4	39.4	39.2	40.2	41.4^#^	49.2
	Cat1	38.2	38.8	38.2	61^#^	53.2
	Cat2	65	65.4	70	78.6^#^	74.6
	Dich1	22.8#	23.6	23.2	24.6	22.2
*N* = 500. corr 0.2 *P*-out < 0.1	Noise	12	11.6^#^	11.6^#^	11.6^#^	10
	Cont4	94.6	94.8^#^	94.8^#^	94.8^#^	99
	Cat1	96.2	98.8	98.6	99.4^#^	100
	Cat2	99.2	100^#^	100^#^	100^#^	100
	Dich1	64.8	65^#^	65^#^	65^#^	69.2
*N* = 500. corr 0.6 *P*-out < 0.1	Noise	10.4	10^#^	10^#^	10^#^	10
	Cont4	82^#^	82^#^	82.2^#^	82^#^	91.2
	Cat1	89.6	93.6	93.2	97.8^#^	98.8
	Cat2	98.4	99.8	99.8	100^#^	100
	Dich1	57.4	58	58	58.4^#^	61.2
*N* = 500. corr 0.2 *P*-out < 0.05	Noise	6.2	6	5.8^#^	6.2	05.2
	Cont4	92	91.8	91.8	92.2^#^	97.8
	Cat1	92.6	96.2	95.4	99^#^	99.8
	Cat2	97.2	99.8	99.8	100^#^	100
	Dich1	52	53	53	53.2^#^	58.8
*N* = 500. corr 0.6 *P*-out < 0.05	Noise	6.4	6.8	6.2^#^	6.2^#^	4.8
	Cont4	39.4	39.2	40.2	41.4^#^	49.2
	Cat1	38.2	38.8	38.2	61^#^	53.2
	Cat2	65	65.4	70^#^	78.6^#^	74.6
	Dich1	22.8^#^	23.6	23.2	24.6	22.2

*N *Number of observations, *corr* Correlation, *P-out* P-value for excluding a variable out of the prognostic model, *Noise *Noise variable, *Cont4* Continuous variable 4, *Cat1* Categorical variable, *Cat2* Categorical variable 2, *Dich1* Dichotomous variable 1, *D1* D1 method, *D2* D2 method, *D3* D3 method, *MPR *Median-P-rule, *comp *analyses in complete dataset (reference values for the pooling methods)

The selection frequency of variables in the complete dataset act as the reference standard: * = reference values for comparison the pooling methods with the complete data; ^#^ = value that is closest to the reference value

### The *P*-values of the selected variables

To compare the P-values of the selected variables with those in the complete datasets, the median of the log-transformed P-values was used. Table 3 shows that for the continuous and dichotomous variables, the pooling methods showed inconsistent results, sometimes the P-values were more close to those obtained in the complete dataset, e.g. the variable ‘Dich1’ in the dataset *N* = 200 with correlation degree 0.2 and a p-out ≤ 0.1 and sometimes not, e.g. the variable ‘Dich1’ in the dataset *N* = 200 with correlation degree 0.6 and a p-out ≤ 0.05. Overall, the P-values for categorical variables obtained with the MPR-method were consistently closer to those obtained in the complete dataset regardless of the sample size, the degree of correlation or the chosen *P*-out.

**Table 3:** *P*-values of the pooled variables after log-transformation and calculation of the median

Dataset	Variables	Pooling Method D1	Pooling Method D2	Pooling Method D3	Pooling Method MPR	Complete Dataset*
*N* = 200 Corr 0.2 *P*-out < 0.1	Noise	-1.309554	-1.3096763^#^	-1.229329	-1.9106885	-1.32294506
	Cont4	-2.012191^#^	-1.8803549	-1.643659	-2.8330669	-2.27920822
	Cat1	-1.930734	-1.8166761	-1.840571	-2.6829073^#^	-2.36633468
	Cat2	-2.150953	-1.9567753	-2.043423	-3.0906292^#^	-2.95750666
	Dich1	-1.755433^#^	-1.746318	-1.696342	-1.8969296	-1.75720332
*N* = 200 Corr 0.6 *P*-out < 0.1	Noise	-1.439446	-1.3603044^#^	-1.285094	-1.7425542	-1.338895
	Cont4	-1.83904^#^	-1.7223828	-1.630539	-2.5040535	-1.95430019
	Cat1	-1.773127^#^	-1.6700756	-1.705565	-2.1858457	-1.92590193
	Cat2	-2.182335	-2.0396509	-2.282796	-2.6709277^#^	-2.54638103
	Dich1	-1.610444	-1.5757474	-1.597061	-1.8110715^#^	-1.72596463
*N* = 200 Corr 0.2 *P*-out < 0.05	Noise	-1.873396^#^	-1.7555967	-1.59476	-2.5543024	-1.90936438
	Cont4	-2.262126^#^	-2.0474303	-1.870487	-3.1615994	-2.53263423
	Cat1	-2.143072	-1.9870785	-2.058752	-2.88706^#^	-2.54509389
	Cat2	-2.434034	-2.1368796	-2.237235	-3.1390634^#^	-3.0642774
	Dich1	-1.984843^#^	-1.9703728	-1.923421	-2.166719	-2.002823
*N* = 200 Corr 0.6 *P*-out < 0.05	Noise	-1.683931^#^	-1.6379195	-1.607315	-2.3293738	-1.86372761
	Cont4	-2.161964^#^	-2.0390992	-1.900202	-2.8912924	-2.374376
	Cat1	-2.078314	-1.920269	-2.029932	-2.4134016^#^	-2.29490206
	Cat2	-2.465616	-2.2333715	-2.489852	-2.8188999^#^	-2.71046546
	Dich1	-1.95608	-1.8382816	-1.854454	-2.0588553^#^	-2.09515079
*N* = 500 Corr 0.2 *P*-out < 0.1	Noise	-1.260527	-1.3075^#^	-1.240598	-1.639111	-1.38193069
	Cont4	-2.936592	-2.7997339	-2.435983	-4.1197571^#^	-4.13946275
	Cat1	-3.194703	-3.6122543	-3.598083	-4.8961963^#^	-5.85087529
	Cat2	-3.713544	-4.3224707	-4.399027	-5.60206^#^	-7.19565311
	Dich1	-1.951632^#^	-1.9122966	-1.833822	-2.0774095	-1.93383386
*N* = 500 Corr 0.6 *P*-out < 0.1	Noise	-1.348488^#^	-1.4031749	-1.401532	-1.9028052	-1.2418635
	Cont4	-2.418733	-2.3622861	-2.145894	-3.3509581^#^	-2.95032364
	Cat1	-2.729985	-2.8352247	-2.758217	-4.0065819^#^	-4.15967193
	Cat2	-4.064997	-4.3178549	-4.49222	-5.1426675^#^	-5.75557227
	Dich1	-1.816346	-1.7937226^#^	-1.764417	-1.9024322	-1.7891513
*N* = 500 Corr 0.2 *P*-out < 0.05	Noise	-1.443512	-1.4313625	-1.388748	-1.8756295^#^	-1.66712132
	Cont4	-3.027566	-2.859925	-2.478627	-4.2321024^#^	-4.1460952
	Cat1	-3.320076	-3.6443612	-3.662341	-4.9232755^#^	-5.84393201
	Cat2	-3.729321	-4.3001623	-4.416825	-5.60206^#^	-7.11509176
	Dich1	-2.16806	-2.1131203^#^	-2.052601	-2.3234185	-2.13648909
*N* = 500 Corr 0.6 *P*-out < 0.05	Noise	-1.683931^#^	-1.6379195	-1.607315	-2.3293738	-1.86372761
	Cont4	-2.161964^#^	-2.0390992	-1.900202	-2.8912924	-2.374376
	Cat1	-2.078314	-1.920269	-2.029932	-2.4134016^#^	-2.29490206
	Cat2	-2.465616	-2.2333715	-2.489852	-2.8188999^#^	-2.71046546
	Dich1	-1.95608	-1.8382816	-1.854454	-2.0588553^#^	-2.09515079

*N* Number of observations, *Corr* Correlation, *P-out* P-value for excluding a variable from the prognostic model, *Noise *Noise variable, *Cont4 *Continuous variable 4, *Cat1* Categorical variable, *Cat2* Categorical variable 2, *Dich1* Dichotomous variable 1, *D1* D1 method, *D2 *D2 method, *D3* D3 method, *MPR* Median-P-rule pooling method, *complete dataset* analyses in complete dataset (reference values for the pooling methods); * = reference values for comparison the pooling methods with the complete data; ^#^ = value that is closest to the reference value

**Fig. 2:**
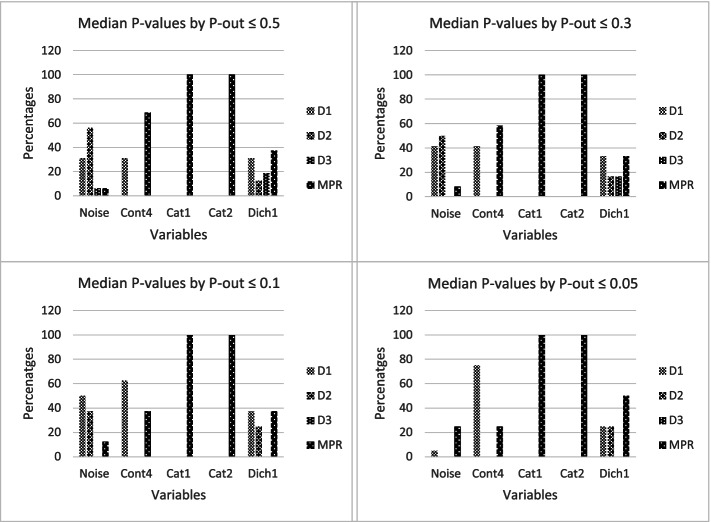
Percentages agreement between the P-values of the selected variables by the different pooling methods and the complete dataset.

Figure 2 shows the percentages of agreement between the *P*-values of the selected variables by the different pooling methods and the complete dataset. It is shown that the MPR-method agreed most with the complete dataset for categorical variables with scores of 100 percent agreement. Evaluating the different levels of p-out, it was clear that for all p-outs the differences between the pooling methods in categorical variables were in favor of the MPR method. For the dichotomous variable the MPR never performed worse than the other pooling methods. For the continuous variables also the D1-method performed reasonably well and performed better than the MPR by stricter *p*-out values.

### The stability of the selected prognostic models

**Fig. 3:**
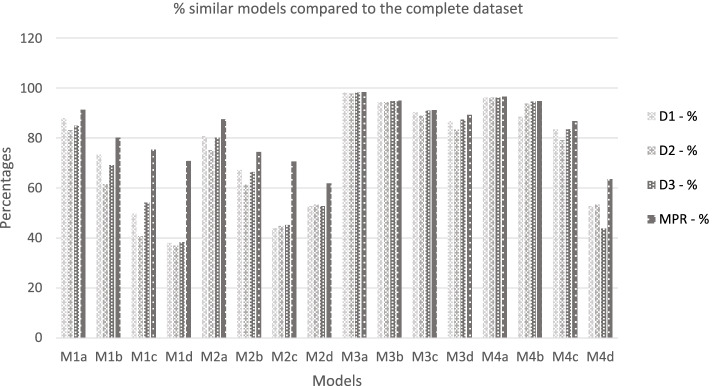
Model selection frequencies of the first ten unique prognostic models from the four pooling methods, quantifying how likely these models were selected compared to the models from the complete dataset

Table 4 shows how the selected models, after applying the different pooling methods, were related to the selected models in the complete dataset. The MPR-method provided models that were more similar to the models in the complete dataset than the other pooling methods. Especially in smaller datasets (*n* = 200), the MPR-method selected more frequently the same prognostic models. The analyses with a *p*-out ≤ 1.0 are not shown because these are not of added value for the development of prediction models. See Table 4 and Fig. 3.

**Table 4:** Comparing selected prognostic models to the developed models in the complete dataset

First 10 unique models	D1(n)	D1(%)	D2(n)	D2(%)	D3(n)	D3(%)	MPR(n)	MPR(%)	Comp (n)
M1^a^									
M1a	425	88.0	402	83.2	410	84.9	441	91.3^#^	483
M1b	329	73.4	276	61.6	310	69.2	359	80.1^#^	448
M1c	178	49.7	146	40.8	194	54.2	270	75.4^#^	358
M1d	110	38.1	107	37.0	111	38.4	205	70.9^#^	289
M2^b^									
M2a	361	80.8	336	75.2	358	80.1	391	87.5^#^	447
M2b	253	67.3	231	61.4	250	66.5	280	74.5^#^	376
M2c	105	43.9	107	44.8	108	45.2	169	70.7^#^	239
M2d	93	52.8	94	53.4	93	52.8	109	61.9^#^	176
M3^c^									
M3a	491	98.2	489	97.8	491	98.2	492	98.4^#^	500
M3b	472	94.4	472	94.4	474	94.8	475	95.0^#^	500
M3c	452	90.4	445	89.0	455	91.0	456	91.2^#^	500
M3d	434	86.8	412	83.4	432	87.4	441	89.3^#^	494
M4^d^									
M4a	481	96.2	481	96.2	481	96.2	483	96.6^#^	500
M4b	439	88.5	465	93.8	469	94.6	470	94.8^#^	496
M4c	401	83.5	380	79.2	401	83.5	416	86.7^#^	480
M4d	93	52.8	94	53.4	77	43.8	112	63.6^#^	176

^a^M1 = model with *n* = 200. correlation degree 0.2; a = *p*-out ≤ 0.5; b = *p*-out ≤ 0.3; c = *p*-out ≤ 0.1; d = *p*-out t ≤ h0.05

^b^M2 = model with *n* = 200. correlation degree 0.6; a = *p*-out ≤ 0.5; b = *p*-out ≤ 0.3; c = *p*-out ≤ 0.1; d = *p*-out ≤ 0.05

^c^M3 = model with *n* = 500. correlation degree 0.2; a = *p*-out ≤ 0.5; b = *p*-out ≤ 0.3; c = *p*-out ≤ 0.1; d = *p*-out ≤ 0.05

^d^M4 = model with *n *= 500. correlation degree 0.6; a = *p*-out ≤ 0.5; b = *p*-out ≤ 0.3; c = *p*-out ≤ 0.1; d = *p*-out ≤ 0.05

*n* Number of observations, *P-out* P-value for excluding variable out of the model, *D1 (n)* Number of developed similar prognostic models as in the complete dataset with the D1-method, *D1(%)* Percentage of similar models as in the complete dataset with the D1-method, *D2 (n)* Number of developed similar prognostic models as in the complete dataset with the D2-method, *D2(%)* Percentage of similar prognostic models as in the complete dataset with the D2-method, *D3 (n)* Number of developed similar prognostic models as in the complete dataset with the D3-method, *D3(%)* Percentage of similar prognostic models as in the complete dataset with the D3-method, *MPR (n)* Number of developed similar prognostic models as in the complete dataset with the MPR-method, *MPR (%)* Percentage of similar prognostic models as in the complete dataset with the MPR-method, *comp (n)* Number of the first ten unique models selected in the BWS-procedure; ^#^ = highest amount of similar unique prognostic models compared to the models from the complete dataset

### Selected variables in the prognostic models in the NHANES-dataset

Table 5 shows that in all imputed NHANES-datasets, the four pooling methods showed a strong similarity in variables selected. Mostly the same variables were selected with comparable p-values. This was in agreement with the findings of the simulation study.

**Table 5:** Selected variables selected in the NHANES-dataset by the four pooling methods

M = 5, P-out < 0.05	*N *= 250				*N* = 300				*N* = 400				*N *= 500				*N *= 1000			
Variables	D1	D2	D3	MPR	D1	D2	D3	MPR	D1	D2	D3	MPR	D1	D2	D3	MPR	D1	D2	D3	MPR
Age	X	X	X	X	X	X	X	X				X	X	X	X	X	X	X	X	X
BMI			X	X					X	X	X	X					X	X	X	X
Pulse																				
BPSysAve																				
BPDiaAve																				
TotChol																				
Gender (Dich)																				
Diabetes (Dich)																				
Race (Cat)																				
Education (Cat)	X	X	X	X	X	X	X	X	X	X	X	X	X	X	X	X	X	X	X	X
Depressed (Cat)																				
LittleInterest (Cat)																X	X		X	X

*M *Number of imputations, *P-out* P-value for excluding a variable from the prognostic model, *N *Number of observations, *D1* D1-method, *D2* D2-method, *D3* D3-method, *MPR* Median P-Rule

## Discussion

### Main findings

In defining a prognostic model after MI with different types of variables including categorical variables, it is important to use an overall test to conclude if a categorical variable is relevant for the model. In the present study Multiply Imputed simulated datasets were used and four selection methods (D1, D2, D3 and MPR) for categorical, dichotomous, and continuous variables in a logistic regression model with a BWS-procedure were evaluated. The selection frequency of variables, P-values of the selected variables and the stability of the selected models were compared with the results from those in the complete dataset. The performance of the MPR is tested in many different conditions and variations and approved to be an easy-to-apply method and to be consistently better (as well in terms of selection frequency as in terms of P-values and the stability of the models) than the other pooling methods in categorical variables in a MI-context. For continuous and dichotomous variables no consistent differences were found between the four pooling methods.

### Comparison with the literature

Eekhout et al. concluded that to obtain correct and powerful pooled *P*-values for significance testing of categorical variables with the MPR, compared to the D1, D2 and D3 methods, the outcome should be omitted from the imputation model [1]. To obtain a powerful significance test for continuous and dichotomous variables with RR after MI, the MI procedure has to include the outcome variable, as was indicated by Moons et al. [19]. We repeated our simulation study in datasets with a sample size of *n* = 500 and *n* = 2000 and an extra categorical variable with five categories under two different conditions: one included the outcome variable in the imputation model, and one excluded the outcome. We observed no differences in the median-P-values of the selected predictor variables or the stability of the selected models. Only the selection frequency of the predictor variables was slightly higher when the outcome was included in the imputation model, but this was identical for all pooling methods. The larger the datasets, the smaller the differences between the four pooling methods. We therefore conclude that for overall significance testing of categorical variables the outcome variable can be included in the imputation model.

Heinze et al. and Wallisch et al. stated that variable selection can compromise stability of a final model. This is an often-ignored problem of data-driven variable selection [4, 18]. Also, Royston and Sauerbrei stated that model stability has to be proven because many different factors influence the stability of the selected models [20, 21]. In our simulation study, we examined the stability of the selected models in Multiply Imputed datasets by repeating each procedure 500 times. An interesting result is that the MPR pooling method resulted in more stable variable selection than the other pooling methods. This result was also reflected in the analyzes in the NHANES-real-world dataset. Austin et al. and Wood et al. stated that variable selection in Multiply Imputed datasets has to be done from the pooled model using RR which is easily done for continuous and dichotomous variables but less straightforward for categorical variables [22, 23]. We distinguished the selection of all types of variables and showed that the MPR-method performed equally well as RR for the continuous and dichotomous variables and better than the D1-, D2- and D3-methods for the categorical variables. The usability of the pooling methods depends on their availability in statistical software. Most software packages do not provide these methods in combination with variable selection and are therefore out of reach for applied researchers. The strength of the MPR-rule is that it can easily be applied in any software package and is not a time-consuming process.

### Strengths and limitations

Our aim was to compare four different selection methods. A strength is that we applied two different ways of pooling and selecting variables: 1. Rubin’s Rules (RR) were applied to pool the continuous and dichotomous variables and the pooling methods D1, D2, D3 and MPR for categorical variables. 2. All variables were pooled by the D1, D2, D3 and MPR method.

No differences were found between those two ways of pooling and selecting variables, i.e., the MPR outperformed all other methods. Another strength is that we used various p-out values to evaluate the behaviour of the pooling methods when the selected models contained variables with a strong or less strong relationship with the outcome as can be found in normal practice. We found that in most scenario’s the MPR method resulted in the most stable models.

Also, a strength is that we, in addition to the study performed by White and Austin et al., carried out many different simulated conditions based on empirical data. We evaluated the selection frequency of the variables, the *P*-values of the selected variables and the model stability of the selected models [22, 23]. Also, we added a noise variable to evaluate if all the methods coped well with this variable. In most of these conditions, the MPR-method was not worse than the other methods. A limitation could be that the simulation study used a small number of covariates than are used in practical data sets. However, the NHANES-dataset contained a mixture of weaker and stronger variables, like in real-world datasets, and the results in the NHANES dataset confirmed what we saw in the simulation study.

Another limitation could be that we used only two different correlation levels in our simulation sets (0.2 and 0.6). However, to set up our simulation study we initially used the paper of Wood, White and Royston [23] about variable selection methods in multiply imputed datasets, that came closest to the aim of our study. They reported a correlation of 0.62 and defined that as a high correlation value. We therefore used a high correlation of 0.6 in our study. We wanted to compare this high correlation with a lower correlation and used the value of 0.2. We think, that by using these values for the correlation, we were well able to test the methods in datasets that are commonly seen in medical studies containing variables with comparable lower and higher correlations. Another limitation may be that we used a fast backward selection procedure to select variables in the complete datasets [24]. It is known that this may be not the most efficient selection method [24, 25]. An alternative may be to use more advanced methods like the least absolute shrinkage and selection operator (LASSO) [25]. However, the LASSO is developed for situations where the number of predictors is much higher than the number of persons. This is not the case in a lot of medical and epidemiological datasets. Another problem with LASSO estimation is its dependence on the scale of the covariates. A solution for this is to apply internal standardization in LASSO software to unit variance before variable selection. After that, the regression coefficients are than back transformed to the original scale. It is however not clear yet if standardization of variables of the type “one size fits all” is the best choice for all modeling purposes. Therefore, using the fast backward selection procedure was the best option to compare the pooled selection methods with a similar selection procedure in the complete datasets [4]. Another limitation could be that we considered all continuous variables as normally distributed while in practice there are also non-linear relationships, so further research will be necessary about the selection of these type of variables in multiply imputed datasets.

## Conclusion

Evaluating four pooling methods (D1, D2, D3 and MPR) for variable selection in multiply imputed datasets for categorical, dichotomous, and continuous variables in logistic regression analyses with a BWS-procedure, the MPR-pooling method performed consistently better than the other methods to select categorical variables in smaller datasets (*N* ≤ 500 participants). The variable selection frequencies, their P-values, the selection frequencies of the prognostic models as well as their stability were more similar to the analyses in the complete datasets using the MPR-method. For continuous and dichotomous variables none of the four pooling methods performed actually better than one of the others. In large datasets there were almost no differences between the four pooling methods. These results were confirmed in the analyzes in a real-world dataset (NHANES). Considering that MPR is the most simple and easy pooling method to use for epidemiologists and applied researchers, we carefully recommend using the MPR-method to pool categorical variables with more than two levels after MI in combination with BWS-procedures. Because MPR never performed worse than the other methods in continuous and dichotomous variables we also advice to use MPR in these types of variables.

## Data Availability

The data that support the findings of this study are available from the web servers of the Amsterdam University Medical Center, but restrictions apply to the availability of these data, which were used under license for the current study, and so are not publicly available. Data are however available from M.W. Heymans, one of the authors, upon reasonable request and with permission of the Amsterdam University Medical center.

## Abbreviations

MI: Multiple Imputation
RR: Rubin’s Rules
BWS: Backward Selection
SE: Standard Error
MPR: Median of the P-values Rule
SD: Standard Deviation
MICE: Multivariate Imputation by Chained Equations
